# Nutritional Therapy for Athletes with Diabetes

**DOI:** 10.3390/jfmk5040083

**Published:** 2020-11-13

**Authors:** Francesca Cannata, Gianluca Vadalà, Luca Ambrosio, Rocco Papalia, Nicola Napoli

**Affiliations:** 1 Department of Endocrinology and Diabetes, Campus Bio-Medico University of Rome, 00128 Rome, Italy; f.cannata@unicampus.it (F.C.); n.napoli@unicampus.it (N.N.); 2 Department of Orthopaedic and Trauma Surgery, Campus Bio-Medico University of Rome, 00128 Rome, Italy; l.ambrosio@unicampus.it (L.A.); r.papalia@unicampus.it (R.P.)

**Keywords:** diabetes, nutritional therapy, macronutrients, athletes, exercise

## Abstract

Diabetes is a worldwide disease also affecting the sports field. The two main forms of diabetes, namely type 1 diabetes (T1D) and type 2 diabetes (T2D), differ in both their pathological and pharmacological characteristics and thus require a distinct nutritional treatment. Diet plays an important role in the management of athletes with diabetes and is crucial to achieving their best performance. This review aims to investigate the objectives of nutritional therapy before, during and after training, in order to improve the best composition of macronutrients during meals. In this review, we provide a brief overview of recent studies about nutritional approaches to people with diabetes for performance optimization and for the control of diabetes-related complications. Thereafter, we discuss the differences between macronutrients and dietary intake before, during and after training. It can be concluded that each sport has particular characteristics in terms of endurance and power, hence demanding a specific energy expenditure and consequent nutritional adjustments. Therefore, the management of athletes with diabetes must be personalized and supported by medical professionals, including a diabetologist, physiologist and a nutritionist.

## 1. Introduction

Many professional athletes, practicing different sports, suffer from diabetes. Currently, there are no specific dietary indications to be applied in the sporting field. Athletes with diabetes should adjust their diet according to the guidelines suggested to the general population [1,2], possibly paying attention to the daily nutritional composition and food amounts, in order to preserve their health and assure a proper performance and strength.

Diabetes mellitus is a chronic disease that includes several physiological dysfunctions [3] with different etiologies: it is characterized by chronic hyperglycemia due to either a defect in insulin secretion and/or functionality. Type 1 diabetes (T1D) is defined by autoimmune pancreatic beta cells destruction due to genetic, immunological, and possibly environmental factors. However, the exact events leading to autoimmune insulitis and T1D development are not yet fully understood [4]. T2D is described by a double defect: insulin is not sufficiently produced to meet the body’s needs, leading to a deficit of insulin secretion, or, the insulin produced does not work adequately thus causing the well-known insulin resistance. The result, in both T1D and T2D, is a persistent increase in blood glucose levels defined as hyperglycemia. T2D is also called non-insulin-dependent because exogenous insulin injection, unlike T1D, is not one of the mainstays of the treatment as it is usually employed only in severe cases. A critical point in diabetes management is the prevention of complications due to persistent hyperglycemia. These mainly include macrovascular and microvascular events, e.g., stroke, acute myocardial infarction, diabetic nephropathy, retinopathy, and neuropathy [5]. Furthermore, diabetic subjects may undergo several changes in the connective tissue, including bone, tendons and cartilage [6]. Therefore, some musculoskeletal disorders are more common in diabetic subjects and include osteoarthritis [7], tendinopathy [8], fractures [9], intervertebral disc degeneration [10,11] and decreased bone mineral density (BMD) [12,13,14].

Nutritional treatment in athletes with diabetes requires an in-depth assessment that passes through several critical points: (1) early evaluation based on the sport practiced, including the type of activity (i.e., strength or endurance) [15,16]; (2) checking of blood glucose [17] followed by optimization of glycemic levels reached during training [1]; (3) scrupulous monitoring of food assumption and antidiabetic treatment [18]. Moreover, it is important to keep a food diary together and to record glycemic levels, especially if dietary changes are necessary [19]. In order to maintain a good glycemic control, it is necessary to have a good balance of carbohydrates (CHO), proteins, and fats [1] distributed in five/six daily meals [20]. The inclusion of snacks is useful in intensive insulin therapy and also in T2D patients [21]. A different approach is possible for T1D patients [22] using a three-meals scheme that needs to be consumed near the insulin injection. Among all the macronutrients, CHO are considered to be mainly responsible for the glycemic increase [21] due to their quick conversion to glucose within an hour of starting the meal [15].

The purpose of this review is to investigate the objectives of nutritional therapy before, during and after training, in order to improve the composition of macronutrients during meals for performance optimization and for the control of diabetes-related complications.

## 2. Nutritional Therapy and T1D

As mentioned above, pharmacological treatment of T1D requires insulin supplementation, therefore athletes affected by T1D must pay attention to the ratio between the prandial insulin dosage [23] and the amount of CHO introduced with food [1]. In common clinical practice, the total amount of CHO should be considered as the main factor in preprandial insulin requirement [24], but it has been shown that the quality of CHO introduced has also a significant impact [25]. The gold standard of nutritional therapy is CHO counting [19] for both intensive insulin therapy and insulin pump [26].

CHO monitoring is crucial (1) to maintain a constant content of CHO in each meal [27]; (2) to learn how to equally substitute foods with the same intake of daily CHO [28] and (3) to adjust insulin therapy to the CHO intake [25].

T1D subjects usually present with a morphotype incline to normal weight, thus not needing caloric restrictions but with a high susceptibility to hypoglycemia compared to individuals with T2D [29]. The objectives of nutritional treatment must provide an adequate caloric intake to treat hypoglycemia in the short and long term. Diet must be designed on the subject and his/her caloric needs in accordance with the sport practiced [29].

The American Diabetes Association (ADA) suggested a diet based on a flexible percentage of CHO, which can vary from 45 to 55% of total calorie intake [25,29,30,31]. Several studies have shown that saccharose intake does not increase glycemic levels more than starch [32], even though the consumption of low glycemic index (GI) food can reduce postprandial glycemia [25,33].

Sugar should not be banned [34], although it is advisable not to exceed 30 g/day [35].

The diet composition of athletes with diabetes must contain other essential macronutrients such as proteins and fats [36] which, if taken correctly, may slightly contribute to the increase in postprandial blood glucose levels [37] and to the need for prandial insulin [38]. Approximately 40–60% of the proteins consumed during the meal are transformed into glucose within four hours after the repast, while fats transformation is the last one to arise for only 10% of the processed fats, occurring several hours after the meal [39]. The current recommendation for people with diabetes establishes a protein intake not exceeding 15% of the total caloric intake [40], considering a daily assumption of 0.80–1 g/day per kg of body weight [41]. Fat content can differ from 30% to 50% of the total caloric intake [42], with polyunsaturated fatty acids (PUFA) ideally accounting for 10% [43]. Olive oil and fish oil consumption should be recommended, as they are considered a major source of monounsaturated fatty acids (MUFA) and omega 3 (ω-3) fatty acids, respectively [36].

However, nutritional recommendations may vary upon the type of exercise practiced by the individual athlete with T1D. Endurance sports (i.e., running, cycling) are considered moderate-intensity activities (40–59% of maximal oxygen consumption (VO_2_ max)) and mainly rely on aerobic metabolism by utilizing muscle glycogen stores and, to a minor extent, circulating free fatty acids (FFAs). When muscle glycogen is depleted, hepatic glycogenolysis and glucose uptake in the skeletal muscle increase upon reduction of insulin secretion and upregulation of released catecholamines. However, athletes with T1D may experience hypoglycemia due to increased insulin absorption, reduced glucagon secretion and impaired catecholaminergic response. Similarly, high-intensity activities (85 to 100% VO_2_ max) are primarily sustained by aerobic metabolism while brief intermittent bouts (typical of team sports, i.e., football, soccer, tennis, volleyball, basketball etc.) activate anaerobic metabolism. Moreover, increased catecholamine release following intermittent sprints may likely result in hyperglycemia during and/or after the exercise [44]. Therefore, food and liquid intake as well as insulin dosage and timing should be carefully adjusted depending on the specific activity in order to maintain muscle and liver glycogen deposits and stable plasma glucose levels [45].

## 3. Nutritional Therapy and T2D

Nutritional therapy planning in athletes with diabetes requires the evaluation of the individual needs, including cultural and personal preferences, the sport practiced, and its duration [34]. According to the guidelines for T2D subjects, relevant components are the fiber amount, glycemic index (GI), and glycemic load (GL) [3,46,47]. Fibers, belonging to the CHO group, are composed of all the macromolecules that are indigestible to gastric enzymes of the digestive system [48]. They are mainly found in food with a vegetal origin, such as fruit, vegetables, whole grains, and legumes [49]. Thanks to the sense of satiety that they offer, fibers may provide significant effects on weight loss [49], maintenance of glycemic levels [50], insulin resistance [51], reduction of cholesterol levels [52] and many others [53]. GI and GL are two important factors for their capacity to influence postprandial glucose concentrations and insulin responses. GI is important for an adequate therapy: it is defined as the incremental area of glycemia after the ingestion of 25–50 g of available CHO and is expressed in percentage of the corresponding area, obtained after consuming a reference food. GL is obtained considering the quantity of ingested CHO for the food GI [47]. As already mentioned, these factors are particularly important for postprandial glycemia, as it is known that insulin secretion after the meal will result in deficit [17,54]. Especially in athletes with T2D, it is certainly advisable to undertake a low-calorie diet to prevent weight gain according to the physical activity level, also considering the significant role of visceral fat in the pathogenesis of diabetes and related complications [55]. This objective must be pursued through a modest reduction of the daily caloric intake, equal to about 300–500 Kcal depending on the sport practiced [56]. As individuals with T2D are mainly middle-aged and overweight, competitive sports are extremely rare among such a population and no specific indication exists regarding the type of sports practiced. Aerobic moderate-intensity activity is advised to promote cardiorespiratory fitness with intermittent resistance bouts to increase muscle strength [45].

The diet quality in terms of food choice is crucial: it has been shown that, in order to achieve weight loss in a short time, two possible dietary approaches could be implemented, namely a hypo-lipidic or a hypo-glucidic diet, but also through a fiber-enriched diet [57].

The CHO content should never drop below 130 g/day in order to avoid gluconeogenesis: sugars may account for 45–60% of the total energy intake, depending on the athlete [20]. Furthermore, the CHO source is also important since many of its characteristics can influence postprandial glucose blood level, such as food type, starch type, maturation, preparation, and processing [58]. The sucrose intake and other added sugars, as they can quickly raise blood sugar level, must be well controlled. Their quantity should never exceed 10% of total energy. It is recommended to use polyols or sweeteners without calories instead of sucrose [59].

Fat intake should not exceed 35% of total calories: total saturated fat must represent about 7–8% of total fats, while MUFA are recommended to be around 0–20%. Trans fatty acids should be avoided, and daily cholesterol intake should not exceed 200 mg [59].

Nutritional recommendations on protein intake should provide a maximum of 15% of the daily total energy. The best intake should not exceed 0.8–1 g/day per kg of body weight [60]. Therefore, protein addition in athletes with T2D may influence an increase in the postprandial insulin response, without increasing glucose concentration [60].

**Figure 1:**
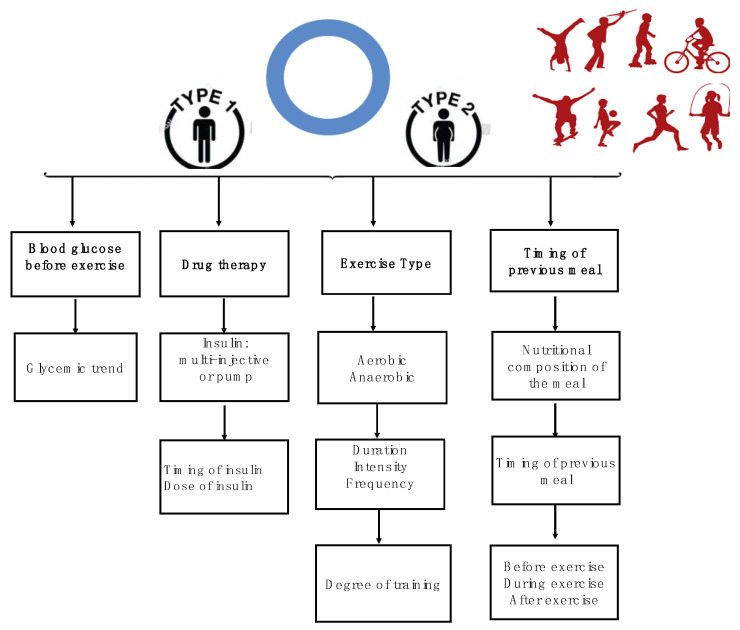
Variables that affect the glycemic response during physical activity in athletes with diabetes.

Major variables involving the glycemic response during physical activity in athletes with T1D and T2D are depicted in Figure 1.

## 4. Training and Competition

### 4.1. Nutrition before Physical Activity

The timing of intake should be 3–4 hours before a sports performance or training. The meal should consist mainly of CHO, include low fat and a moderate protein content, providing the essential fluids to assure an optimal degree of hydration [61]. To avoid gastrointestinal disorders, the meal should only consist of food that is well tolerated by the athlete. A small amount of CHO can also be taken up to one hour before exercise [16]. The consumption of balanced meals can improve athletic performance and restore sources of muscle and liver glycogen [60]. Ideally, it is desirable to consume low GI food before training. However, scientific evidence on athletes with diabetes has not been able to demonstrate a clear advantage of low GI pre-training meals. According to common practice, an athlete with diabetes may need to consume 15 g of complex CHO 15–30 min before a moderate workout of approximately 45 min [62].

Blood glucose should be strictly monitored before exercise. If low blood glucose (<100 mg/dL) is detected, extra CHO should be taken before beginning the activity [63]. If training is regular, blood glucose control will be easier. Snacks included in the daily diet plan facilitate exercise needs and any usual doses of insulin. It is essential to assess blood glucose through continuous glucose monitoring (CGM) [64], which is necessary to optimize possible dietary changes throughout physical activity, especially in insulin-treated patients. A technique used by athletes to increase the sources of glycogen in case of long-term sporting events is a GL [65]. It depends on the availability of insulin and requires a cautious control of glycemia [66]. It is necessary to monitor insulin administration to keep up with the CHO consumption [14], considering the possible effects of exercise reduction of the athletes before the competition. Hence, glycemic supervision is essential for GL. The main issues regarding nutrition before training are summarized in Table 1.

### 4.2. Nutrition during Physical Activity

Eating during exercise depends on training length and intensity. Generally, CHO supplements should be assumed only when the length of training exceeds 1 h or during competitive sports events [66]. This is particularly important when the athlete has not properly consumed any food nor liquids before training, or if the athlete is engaged in extreme climate/weather conditions [67]. Considering the higher hypoglycemic risk compared to non-diabetic competitors, athletes with diabetes should take 30–60 g of CHO per hour, possibly consumed every 15–30 min [68]. Extra CHO should be consumed frequently and in small quantities, both in liquid and solid forms [69]. Liquid CHO have the advantage of being assimilated more rapidly, and also lead to a reintegration of body water. Gastric emptying is highly influenced by the content of sugars. Indeed, solutions with ≤8% of CHO have the same gastric emptying speed of water, whereas solutions containing >10% of CHO may increase osmotic water absorption across the intestinal lumen with the risk of gastrointestinal disorders [18]. Fruit juices and most of the available soft beverages contain approximately 12% of CHO and should be diluted with water. Additional options include CHO gel, fruits, and protein bars [70]. The main issues regarding nutrition during training are summarized in Table 1.

### 4.3. Nutrition after Physical Activity

The main goal after training is to provide for adequate liquids, electrolytes, energy, and CHO to replace muscle glycogen and guarantee a rapid recovery [71].

**Figure 2:**
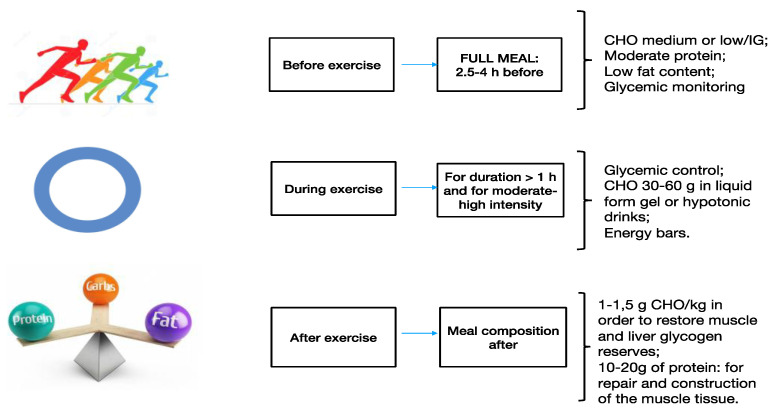
Macronutrient composition of meal before, during, and after physical activity in athletes with diabetes.

A CHO intake of approximately 1–1.5 g/kg of body weight during the first 30 min after physical activity and every 2 h for 4–6 h will be enough to reconstitute liver and muscle glycogen sources [72]. Similarly, protein assumption after a workout will supply the amino acids for muscular tissue synthesis and repair [73,74]. Nutritional strategies during recovery are meant to replace the substrates and liquids that have been used during the training and are the same for athletes without diabetes [75]. However, it is important to consider the increased insulin sensitivity caused by physical activity, both in amateurs and professional athletes with diabetes which continues even after exercise [76]. Therefore, the risk of hypoglycemia endures for several hours, and hypoglycemia may occur up to 4–48 h after training and competition [77]. In order to prevent late hypoglycemia, a satisfying quantity of CHO should be assumed before, during, and after physical activity. Finally, it is necessary to reduce the insulin dosage after training, and it is essential to frequently monitor glycemia levels after training as well [66]. Unfortunately, late hypoglycemia often occurs at night, and if it happens regularly this can lead to tiredness in athletes [69]. The macronutrient composition of a meal before, during, and after training in athletes with diabetes is described in Figure 2. The main issues regarding nutrition after training are summarized in Table 1.

## 5. Discussion

The nutritional approach in the diet of T1D and T2D athletes should strictly adhere to the principles recommended for diabetes treatment, providing the energy and nutritional needs and water loss related to physical activity [20]. The Mediterranean model, universally recognized and recommended by international scientific societies and guidelines for primary and secondary prevention of chronic and degenerative diseases, has been shown to improve the glycemic and lipid profile in diabetic patients, reducing diabetes incidence by 52% compared to other low-fat diets [78]. CHO-proteins ratio should be moderately increased compared to those who do not practice sport [2]. A balanced diet based on vegetables and animal products is able to meet the need for essential amino acids as well as minerals and vitamins. Supplements based on energy bars and energy drinks should only be considered in sports activities where exercise is intense and long-lasting and energy expenditure is very high [79]. Many factors influence the glycemic response to physical activity, making it impossible to provide nutritional and insulin dosage indications that can apply to all subjects. Among these factors, exercise intensity, duration, and type, as well as the training level of the individual, the nutritional status in terms of glycogen deposits, time relationships with previous food intake, and macronutrient composition of food introduced must be taken into account [80]. Furthermore, diabetes type and severity, control of glucometabolic state, blood sugar levels before physical activity, the type of insulin or other hypoglycemic drugs, and their last assumption related to sport practice are relevant. Therefore, for a safe and effective practice of physical activity for competitive purposes, strict attention to signs and symptoms predictive of hypoglycemia or other nutritional deficits with intensive monitoring of glycemia is necessary.

## 6. Conclusions

Athletes with diabetes need to follow a diet that is similar to the one recommended for non-athletic diabetic individuals: caloric and energetic requirements must always be satisfied, with a cautious reintegration of hydric and nutrients loss after exercise. It is important to detect early and recognize signs of nutritional deficiency with intensive control of glycemia and body weight, in order to practice physical activity in a safe and efficient way. The continuous assistance of professional experts is required to optimize the management of athletes with diabetes for long-term safe and effective dietary therapy.

## Figures and Tables

**Table 1:** Main issues regarding nutrition before, during, and after training.

Before Training	During Training	After Training
Considering exercise type, length, intensity. Timing according to meals and glycemic trend.	Monitoring glycemia levels, especially if the activity exceeds 1 h.	Carbohydrates (CHO) are necessary to reconstitute muscular and liver glycogen sources. Liquids and sodium are necessary for rehydration.
Tools for CGM, equipped with increase and decrease glycemic indicators.	Consuming small quantities of CHO with different absorption speeds.	Proteins are necessary to repair and assemble muscular tissue, in response to the workout.
Planning glycemic controls every 45 min–1 h.	Checking glycemia approximately every 30 min after training to establish the appropriate CHO requirement.	Awareness of the risk of late hypoglycemia > up to 48 h later.

## References

[1] Mann J.I., De Leeuw I., Hermansen K., Karamanos B., Karlström B., Katsilambros N., Riccardi G., Rivellese A.A., Rizkalla S., Slama G. (2004). Evidence-based nutritional approaches to the treatment and prevention of diabetes mellitus. Nutr. Metab. Cardiovasc. Dis..

[2] Marathe P.H., Gao H.X., Close K.L. (2017). American Diabetes Association Standards of Medical Care in Diabetes 2017. J. Diabetes.

[3] Bantle J.P., Wylie-Rosett J., Albright A.L., Apovian C.M., Clark N.G., Franz M.J., Hoogwerf B.J., Lichtenstein A.H., Mayer-Davis E., Mooradian A.D. (2006). Nutrition recommendations and interventions for diabetes--2006: A position statement of the American Diabetes Association. Diabetes Care.

[4] van Belle T.L., Coppieters K.T., von Herrath M.G. (2011). Type 1 diabetes: Etiology, immunology, and therapeutic strategies. Physiol. Rev..

[5] IDF Diabetes Atlas. http://www.diabetesatlas.org.

[6] Piccoli A., Cannata F., Strollo R., Pedone C., Leanza G., Russo F., Greto V., Isgrò C., Quattrocchi C.C., Massaroni C. Sclerostin Regulation, Microarchitecture, and Advanced Glycation End-Products in the Bone of Elderly Women With Type 2 Diabetes. J. Bone Miner Res..

[7] Cannata F., Vadala G., Ambrosio L., Napoli N., Papalia R., Denaro V., Pozzilli P. (2020). Osteoarthritis and type 2 diabetes: From pathogenetic factors to therapeutic intervention. Diabetes Metab. Res. Rev..

[8] Cannata F., Vadalà G., Ambrosio L., Napoli N., Papalia R., Denaro V., Pozzilli P. The impact of type 2 diabetes on the development of tendinopathy. Diabetes Metab. Res. Rev..

[9] Hothersall E.J., Livingstone S.J., Looker H.C., Ahmed S.F., Cleland S., Leese G.P., Lindsay R.S., McKnight J., Pearson D., Philip S. (2014). Contemporary risk of hip fracture in type 1 and type 2 diabetes: A national registry study from Scotland. J. Bone Miner. Res..

[10] Cannata F., Vadala G., Ambrosio L., Fallucca S., Napoli N., Papalia R., Pozzilli P., Denaro V. (2020). Intervertebral disc degeneration: A focus on obesity and type 2 diabetes. Diabetes Metab. Res. Rev..

[11] Russo F., Ambrosio L., Ngo K., Vadala G., Denaro V., Fan Y., Sowa G., Kang J.D., Vo N. (2019). The Role of Type I Diabetes in Intervertebral Disc Degeneration. Spine.

[12] Agius R., Galea R., Fava S. (2016). Bone mineral density and intervertebral disc height in type 2 diabetes. J. Diabetes Complicat..

[13] Napoli N., Conte C., Pedone C., Strotmeyer E.S., Barbour K.E., Black D.M., Samelson E.J., Schwartz A.V. (2019). Effect of Insulin Resistance on BMD and Fracture Risk in Older Adults. J. Clin. Endocrinol. Metab..

[14] Armamento-Villareal R., Aguirre L., Waters D.L., Napoli N., Qualls C., Villareal D.T. (2020). Effect of Aerobic or Resistance Exercise, or Both, on Bone Mineral Density and Bone Metabolism in Obese Older Adults While Dieting: A Randomized Controlled Trial. J. Bone Miner. Res..

[15] Kerksick C.M., Wilborn C.D., Roberts M.D., Smith-Ryan A., Kleiner S.M., Jäger R., Collins R., Cooke M., Davis J.N., Galvan E. (2018). ISSN exercise & sports nutrition review update: Research & recommendations. J. Int. Soc. Sports Nutr..

[16] Burke L.M., Hawley J.A., Wong S.H., Jeukendrup A.E. (2011). Carbohydrates for training and competition. J. Sports Sci..

[17] Riccardi G., Rivellese A.A., Giacco R. (2008). Role of glycemic index and glycemic load in the healthy state, in prediabetes, and in diabetes. Am. J. Clin. Nutr..

[18] Mason W.L., McConell G., Hargreaves M. (1993). Carbohydrate ingestion during exercise: Liquid vs solid feedings. Med. Sci. Sports Exerc..

[19] Feinman R.D., Pogozelski W.K., Astrup A., Bernstein R.K., Fine E.J., Westman E.C., Accurso A., Frassetto L., Gower B.A., McFarlane S.I. (2019). Corrigendum to “Dietary carbohydrate restriction as the first approach in diabetes management: Critical review and evidence base” [Nutrition 31 (2015) 1-13]. Nutrition.

[20] Mann J.I., Riccardi G. (2004). Evidence-based European guidelines on diet and diabetes. Nutr. Metab. Cardiovasc. Dis..

[21] Ha V., Viguiliouk E., Kendall C.W.C., Balachandran B., Jenkins D.J.A., Kavsak P.A., Sievenpiper J.L. (2017). Effect of a low glycemic index diet versus a high-cereal fibre diet on markers of subclinical cardiac injury in healthy individuals with type 2 diabetes mellitus: An exploratory analysis of a randomized dietary trial. Clin. Biochem..

[22] Perrotti N., Santoro D., Genovese S., Giacco A., Rivellese A., Riccardi G. (1984). Effect of digestible carbohydrates on glucose control in insulin-dependent diabetic patients. Diabetes Care.

[23] Atkinson M.A., Eisenbarth G.S. (2001). Type 1 diabetes: New perspectives on disease pathogenesis and treatment. Lancet.

[24] Yardley J.E., Sigal R.J., Kenny G.P., Riddell M.C., Lovblom L.E., Perkins B.A. (2013). Point accuracy of interstitial continuous glucose monitoring during exercise in type 1 diabetes. Diabetes Technol. Ther..

[25] Nathan D.M., Group D.E.R. (2014). The diabetes control and complications trial/epidemiology of diabetes interventions and complications study at 30 years: Overview. Diabetes Care.

[26] Lennerz B.S., Barton A., Bernstein R.K., Dikeman R.D., Diulus C., Hallberg S., Rhodes E.T., Ebbeling C.B., Westman E.C., Yancy W.S. (2018). Management of Type 1 Diabetes with a Very Low-Carbohydrate Diet. Pediatrics.

[27] Riddell M.C., Gallen I.W., Smart C.E., Taplin C.E., Adolfsson P., Lumb A.N., Kowalski A., Rabasa-Lhoret R., McCrimmon R.J., Hume C. (2017). Exercise management in type 1 diabetes: A consensus statement. Lancet Diabetes Endocrinol..

[28] Bryden K.S., Neil A., Mayou R.A., Peveler R.C., Fairburn C.G., Dunger D.B. (1999). Eating habits, body weight, and insulin misuse. A longitudinal study of teenagers and young adults with type 1 diabetes. Diabetes Care.

[29] Ramtoola S., Nyeland M.E., Jacobsen J., Ploug U.J., Kragh N., Zimmermann E. (2019). Characteristics of newly diagnosed adults with type 1 diabetes in the UK and evolution of glycaemic control, body mass index and Charlson comorbidity index over the first 5 years after diagnosis. Prim. Care Diabetes.

[30] Burke L.M., Castell L.M., Casa D.J., Close G.L., Costa R.J.S., Desbrow B., Halson S.L., Lis D.M., Melin A.K., Peeling P. (2019). International Association of Athletics Federations Consensus Statement 2019: Nutrition for Athletics. Int. J. Sport Nutr. Exerc. Metab..

[31] Carracher A.M., Marathe P.H., Close K.L. (2018). European Association for the Study of Diabetes 2017. J. Diabetes.

[32] Johnson R.J., Murray R. (2010). Fructose, exercise, and health. Curr. Sports Med. Rep..

[33] Campbell M.D., Walker M., King D., Gonzalez J.T., Allerton D., Stevenson E.J., Shaw J.A., West D.J. (2016). Carbohydrate Counting at Meal Time Followed by a Small Secondary Postprandial Bolus Injection at 3 Hours Prevents Late Hyperglycemia, Without Hypoglycemia, After a High-Carbohydrate, High-Fat Meal in Type 1 Diabetes. Diabetes Care.

[34] Nielsen J.V., Gando C., Joensson E., Paulsson C. (2012). Low carbohydrate diet in type 1 diabetes, long-term improvement and adherence: A clinical audit. Diabetol. Metab. Syndr..

[35] Marquet L.A., Hausswirth C., Molle O., Hawley J.A., Burke L.M., Tiollier E., Brisswalter J. (2016). Periodization of Carbohydrate Intake: Short-Term Effect on Performance. Nutrients.

[36] Fritzen A.M., Lundsgaard A.M., Kiens B. (2019). Dietary Fuels in Athletic Performance. Annu. Rev. Nutr..

[37] Bell K.J., Smart C.E., Steil G.M., Brand-Miller J.C., King B., Wolpert H.A. (2015). Impact of fat, protein, and glycemic index on postprandial glucose control in type 1 diabetes: Implications for intensive diabetes management in the continuous glucose monitoring era. Diabetes Care.

[38] Galassetti P., Tate D., Neill R.A., Richardson A., Leu S.Y., Davis S.N. (2006). Effect of differing antecedent hypoglycemia on counterregulatory responses to exercise in type 1 diabetes. Am. J. Physiol. Endocrinol. Metab..

[39] Spriet L.L. (2014). New insights into the interaction of carbohydrate and fat metabolism during exercise. Sports Med..

[40] Iafusco D. (2006). Diet and physical activity in patients with type 1 diabetes. Acta Biomed..

[41] Hector A.J., Phillips S.M. (2018). Protein Recommendations for Weight Loss in Elite Athletes: A Focus on Body Composition and Performance. Int. J. Sport Nutr. Exerc. Metab..

[42] Fritzen A.M., Lundsgaard A.M., Jeppesen J.F., Sjoberg K.A., Hoeg L.D., Deleuran H.H., Wojtaszewski J.F.P., Richter E.A., Kiens B. (2019). Fatty acid type-specific regulation of SIRT1 does not affect insulin sensitivity in human skeletal muscle. FASEB J..

[43] Jenner S.L., Buckley G.L., Belski R., Devlin B.L., Forsyth A.K. (2019). Dietary Intakes of Professional and Semi-Professional Team Sport Athletes Do Not Meet Sport Nutrition Recommendations-A Systematic Literature Review. Nutrients.

[44] Horton W.B., Subauste J.S. (2016). Care of the Athlete with Type 1 Diabetes Mellitus: A Clinical Review. Int. J. Endocrinol. Metab..

[45] Hornsby W.G., Chetlin R.D. (2005). Management of Competitive Athletes with Diabetes. Diabetes Spectrum.

[46] Colberg S.R., Albright A.L., Blissmer B.J., Braun B., Chasan-Taber L., Fernhall B., Regensteiner J.G., Rubin R.R., Sigal R.J., American College of Sports M. (2010). Exercise and type 2 diabetes: American College of Sports Medicine and the American Diabetes Association: Joint position statement. Exercise and type 2 diabetes. Med. Sci. Sports Exerc..

[47] Thomas D., Elliott E.J. (2009). Low glycaemic index, or low glycaemic load, diets for diabetes mellitus. Cochrane Database Syst. Rev..

[48] Slavin J. (2013). Fiber and prebiotics: Mechanisms and health benefits. Nutrients.

[49] Palacios O.M., Kramer M., Maki K.C. (2019). Diet and prevention of type 2 diabetes mellitus: Beyond weight loss and exercise. Expert Rev. Endocrinol. Metab..

[50] Derosa G., Limas C.P., Macias P.C., Estrella A., Maffioli P. (2014). Dietary and nutraceutical approach to type 2 diabetes. Arch. Med. Sci..

[51] Weickert M.O., Pfeiffer A.F.H. (2018). Impact of Dietary Fiber Consumption on Insulin Resistance and the Prevention of Type 2 Diabetes. J. Nutr..

[52] Sima P., Vannucci L., Vetvicka V. (2018). beta-glucans and cholesterol (Review). Int. J. Mol. Med..

[53] Aleixandre A., Miguel M. (2016). Dietary fiber and blood pressure control. Food Funct..

[54] Jenkins D.J., Kendall C.W., McKeown-Eyssen G., Josse R.G., Silverberg J., Booth G.L., Vidgen E., Josse A.R., Nguyen T.H., Corrigan S. (2008). Effect of a low-glycemic index or a high-cereal fiber diet on type 2 diabetes: A randomized trial. JAMA.

[55] Colberg S.R., Hill L.C., Parson H.K., Thomas K.S., Vinik A.I. (2010). Aerobic training increases skin perfusion by a nitric oxide mechanism in type 2 diabetes. Diabetes Metab. Syndr. Obes..

[56] Madden K.M. (2013). Evidence for the benefit of exercise therapy in patients with type 2 diabetes. Diabetes Metab. Syndr. Obes..

[57] Nordmann A.J., Nordmann A., Briel M., Keller U., Yancy W.S., Brehm B.J., Bucher H.C. (2006). Effects of low-carbohydrate vs low-fat diets on weight loss and cardiovascular risk factors: A meta-analysis of randomized controlled trials. Arch. Intern. Med..

[58] Coggan A.R., Coyle E.F. (1991). Carbohydrate ingestion during prolonged exercise: Effects on metabolism and performance. Exerc. Sport Sci. Rev..

[59] Dyson P.A., Kelly T., Deakin T., Duncan A., Frost G., Harrison Z., Khatri D., Kunka D., McArdle P., Mellor D. (2011). Diabetes UK evidence-based nutrition guidelines for the prevention and management of diabetes. Diabet. Med..

[60] Ley S.H., Hamdy O., Mohan V., Hu F.B. (2014). Prevention and management of type 2 diabetes: Dietary components and nutritional strategies. Lancet.

[61] Trumbo P., Schlicker S., Yates A.A., Poos M. (2002). Dietary reference intakes for energy, carbohydrate, fiber, fat, fatty acids, cholesterol, protein and amino acids. J. Am. Diet. Assoc..

[62] Sherman W.M., Costill D.L., Fink W.J., Miller J.M. (1981). Effect of exercise-diet manipulation on muscle glycogen and its subsequent utilization during performance. Int. J. Sports Med..

[63] Neufer P.D., Costill D.L., Flynn M.G., Kirwan J.P., Mitchell J.B., Houmard J. (1987). Improvements in exercise performance: Effects of carbohydrate feedings and diet. J. Appl. Physiol..

[64] Burke L.M., Jeukendrup A.E., Jones A.M., Mooses M. (2019). Contemporary Nutrition Strategies to Optimize Performance in Distance Runners and Race Walkers. Int. J. Sport Nutr. Exerc. Metab..

[65] Nathan D.M., Madnek S.F., Delahanty L. (1985). Programming pre-exercise snacks to prevent post-exercise hypoglycemia in intensively treated insulin-dependent diabetics. Ann. Intern. Med..

[66] Mondazzi L., Arcelli E. (2009). Glycemic index in sport nutrition. J. Am. Coll. Nutr..

[67] Cermak N.M., van Loon L.J. (2013). The use of carbohydrates during exercise as an ergogenic aid. Sports Med..

[68] Learsi S.K., Ghiarone T., Silva-Cavalcante M.D., Andrade-Souza V.A., Ataide-Silva T., Bertuzzi R., de Araujo G.G., McConell G., Lima-Silva A.E. (2019). Cycling time trial performance is improved by carbohydrate ingestion during exercise regardless of a fed or fasted state. Scand. J. Med. Sci. Sports.

[69] De Bock K., Derave W., Eijnde B.O., Hesselink M.K., Koninckx E., Rose A.J., Schrauwen P., Bonen A., Richter E.A., Hespel P. (2008). Effect of training in the fasted state on metabolic responses during exercise with carbohydrate intake. J. Appl. Physiol..

[70] Wilson P.B., Ingraham S.J. (2015). Glucose-fructose likely improves gastrointestinal comfort and endurance running performance relative to glucose-only. Scand. J. Med. Sci. Sports.

[71] Jager R., Kerksick C.M., Campbell B.I., Cribb P.J., Wells S.D., Skwiat T.M., Purpura M., Ziegenfuss T.N., Ferrando A.A., Arent S.M. (2017). International Society of Sports Nutrition Position Stand: Protein and exercise. J. Int. Soc. Sports Nutr..

[72] Gonzalez J.T., Fuchs C.J., Betts J.A., van Loon L.J. (2016). Liver glycogen metabolism during and after prolonged endurance-type exercise. Am. J. Physiol. Endocrinol. Metab..

[73] Wallis G.A., Hulston C.J., Mann C.H., Roper H.P., Tipton K.D., Jeukendrup A.E. (2008). Postexercise muscle glycogen synthesis with combined glucose and fructose ingestion. Med. Sci. Sports Exerc..

[74] Pennings B., Koopman R., Beelen M., Senden J.M., Saris W.H., van Loon L.J. (2011). Exercising before protein intake allows for greater use of dietary protein-derived amino acids for de novo muscle protein synthesis in both young and elderly men. Am. J. Clin. Nutr..

[75] van Loon L.J., Kruijshoop M., Verhagen H., Saris W.H., Wagenmakers A.J. (2000). Ingestion of protein hydrolysate and amino acid-carbohydrate mixtures increases postexercise plasma insulin responses in men. J. Nutr..

[76] Stocks B., Dent J.R., Ogden H.B., Zemp M., Philp A. (2019). Postexercise skeletal muscle signaling responses to moderate- to high-intensity steady-state exercise in the fed or fasted state. Am. J. Physiol. Endocrinol. Metab..

[77] Beelen M., Burke L.M., Gibala M.J., van Loon L.J. (2010). Nutritional strategies to promote postexercise recovery. Int. J. Sport Nutr. Exerc. Metab..

[78] Salas-Salvado J., Bullo M., Babio N., Martinez-Gonzalez M.A., Ibarrola-Jurado N., Basora J., Estruch R., Covas M.I., Corella D., Aros F. (2011). Reduction in the incidence of type 2 diabetes with the Mediterranean diet: Results of the PREDIMED-Reus nutrition intervention randomized trial. Diabetes Care.

[79] Bell K.J., Barclay A.W., Petocz P., Colagiuri S., Brand-Miller J.C. (2014). Efficacy of carbohydrate counting in type 1 diabetes: A systematic review and meta-analysis. Lancet Diabetes Endocrinol..

[80] Augustin L.S., Kendall C.W., Jenkins D.J., Willett W.C., Astrup A., Barclay A.W., Bjorck I., Brand-Miller J.C., Brighenti F., Buyken A.E. (2015). Glycemic index, glycemic load and glycemic response: An International Scientific Consensus Summit from the International Carbohydrate Quality Consortium (ICQC). Nutr. Metab. Cardiovasc. Dis..

